# The pediatric gut bacteriome and virome in response to SARS-CoV-2 infection

**DOI:** 10.3389/fcimb.2024.1335450

**Published:** 2024-01-22

**Authors:** Antonia Piazzesi, Stefania Pane, Federica Del Chierico, Lorenza Romani, Andrea Campana, Paolo Palma, Lorenza Putignani

**Affiliations:** ^1^ Unit of Human Microbiome, Bambino Gesù Children’s Hospital, IRCCS, Rome, Italy; ^2^ Unit of Microbiomics, Bambino Gesù Children’s Hospital, IRCCS, Rome, Italy; ^3^ Infectious Diseases Unit, Bambino Gesù Children’s Hospital, IRCCS, Rome, Italy; ^4^ Department of Pediatrics, Bambino Gesù Children’s Hospital, IRCCS, Rome, Italy; ^5^ Unit of Clinical Immunology and Vaccinology, Bambino Gesù Children’s Hospital, IRCCS, Rome, Italy; ^6^ Chair of Pediatrics, Department of Systems Medicine, University of Rome “Tor Vergata”, Rome, Italy; ^7^ Unit of Microbiomics and Unit of Human Microbiome, Bambino Gesù Children’s Hospital, IRCCS, Rome, Italy

**Keywords:** gut microbiota, SARS-CoV-2, COVID-19, pediatric, shotgun sequencing, bacteriome, virome, antibiotics

## Abstract

**Introduction:**

Since the beginning of the SARS-CoV-2 pandemic in early 2020, it has been apparent that children were partially protected from both infection and the more severe forms of the disease. Many different mechanisms have been proposed to explain this phenomenon, including children’s frequent exposure to other upper respiratory infections and vaccines, and which inflammatory cytokines they are more likely to produce in response to infection. Furthermore, given the presence of SARS-CoV-2 in the intestine and its ability to infect enterocytes, combined with the well described immunomodulatory capabilities of the microbiome, another potential contributing factor may be the presence of certain protective microbial members of the gut microbiota (GM).

**Methods:**

We performed shotgun metagenomic sequencing and profiled both the bacteriome and virome of the GM of pediatric SARS-CoV-2 patients compared to healthy, age-matched subjects.

**Results:**

We found that, while pediatric patients do share some pro-inflammatory microbial signatures with adult patients, they also possess a distinct microbial signature of protective bacteria previously found to be negatively correlated with SARS-CoV-2 infectivity and COVID-19 severity. COVID-19 was also associated with higher fecal *Cytomegalovirus* load, and with shifts in the relative abundances of bacteriophages in the GM. Furthermore, we address how the preventative treatment of COVID-19 patients with antibiotics, a common practice especially in the early days of the pandemic, affected the bacteriome and virome, as well as the abundances of antimicrobial resistance and virulence genes in these patients.

**Discussion:**

To our knowledge, this is the first study to address the bacteriome, virome, and resistome of pediatric patients in response to COVID-19 and to preventative antibiotics use.

## Introduction

Since the identification of the first human subjects infected by the severe acute respiratory syndrome coronavirus-2 (SARS-CoV-2) a few short years ago, coronavirus disease 19 (COVID-19) rapidly spread across the world, infecting hundreds of millions of people and causing the largest global health crisis in living memory. Since the beginning of the pandemic, clinicians and epidemiologists noticed that children seemed to be more protected from the virus than their adult family members, both in terms of becoming infected and in developing the more severe manifestations of the disease (Massalska and Gober, 2021; Chou et al., 2022; Kalyanaraman and Anderson, 2022). While this protection from severe COVID-19 was by no means universal, especially in children with underlying pathological conditions, there was an undeniable pattern of fewer hospitalizations and fewer deaths among pediatric patients infected with SARS-CoV-2 (Massalska and Gober, 2021; Chou et al., 2022; Kalyanaraman and Anderson, 2022).

Many different biological processes are thought to confer this partial protection to pediatric COVID-19 patients. From an immunological perspective, studies have indicated that children produce antibodies with higher virus-neutralizing capabilities in response to SARS-CoV-2 infection (Garrido et al., 2021; Massalska and Gober, 2021; Yang et al., 2021; Chou et al., 2022), which has led some researchers to hypothesize that children’s frequent exposure to respiratory viruses and vaccines has left their immune system primed for a robust response to SARS-CoV-2 infection (Massalska and Gober, 2021). Furthermore, children naturally produce more anti-inflammatory cytokines such as IL-10 upon immune activation, while IL-6, the cytokine primarily responsible for the cytokine storm at the root of many COVID-19-related deaths, is produced less in children (Lingappan et al., 2020; Massalska and Gober, 2021). However, despite being a disease of the upper respiratory system, recent evidence has led to the hypothesis that the human gut microbiome could also aid in the protection against the more severe forms of COVID-19.

SARS-CoV-2 infection is achieved by viral interaction with Angiotensin-converting enzyme 2 (ACE2) receptor and the transmembrane serine protease 2 (TMPRSS2) on host cells (Beyerstedt et al., 2021). While *ACE2* and *TMPRSS2* are highly co-expressed in the lungs, where SARS-CoV-2 infection does the most damage, they are also highly expressed in the gastrointestinal tract (Zhang et al., 2020). Consistently, a large percentage of patients infected with SARS-CoV-2 also report gastrointestinal symptoms, and the virus has been detected in stool samples even after the infection has been cleared by the upper respiratory system (Groff et al., 2021). Furthermore, it has been experimentally confirmed that SARS-CoV-2 actively replicates in human enterocytes (Lamers et al., 2020). Given the frequent presence of SARS-CoV-2 in the intestine, it is perhaps unsurprising that COVID-19 has been associated with the imbalance of the gut microbiota (GM) physiology and ecology, commonly referred to as dysbiosis. Mechanistically, it has been proposed that SARS-CoV-2 provokes dysbiosis by both triggering intestinal inflammation and by dysregulating ACE2, both of which have been shown to alter the microbial composition of the gut (Hashimoto et al., 2012; Guimarães Sousa et al., 2022; Li et al., 2022).

However, this influence can go both ways, and there are many ways that the GM can either aid or inhibit viral infectivity. For example, several *Bacteroides* species, such as *Bacteroides dorei, Bacteroides massiliensis, Bacteroides ovatus* and *Bacteroides thetaiotaomicron*, can inhibit *ACE2* expression, thus affecting SARS-CoV-2 infectivity (Zuo et al., 2020). Other bacterial species have immunomodulatory functions, and thus can affect COVID-19 progression even beyond a direct inhibition of the virus within the gut. For example, *Lactobacillus plantarum* and *Pediococcus acidilactici* have shown some promise as both a treatment and as a prophylaxis against severe COVID-19 by modulating the host’s immune system and inducing a protective cytokine response (Gutiérrez-Castrellón et al., 2022; Kageyama et al., 2022). *Faecalibacterium prausnitzii*, whose abundance has been found to be negatively correlated with COVID-19 severity in adult patients (Zuo et al., 2020; Yeoh et al., 2021), has been found to both downregulate *ACE2 in vitro* and have immunomodulatory effects *in vivo*, leading to many considering it to be an attractive candidate for next generation probiotics (Cyprian et al., 2021; Ahmadi Badi et al., 2022; Martín et al., 2023).

Historically, the vast majority of GM studies have focused on the bacterial kingdom and its undeniably fundamental role in human health and disease. However, the GM is a highly complex ecosystem composed not only of bacteria, but also of commensal viruses, fungi and archaea, which have the potential to participate in trans-kingdom interactions and influence human health. In fact, recent evidence has uncovered tens of thousands of viral species inhabiting the human gut, making it a substantial player in the human GM with a diversity to rival even that of the bacterial kingdom (Camarillo-Guerrero et al., 2021; Cao et al., 2022; Pargin et al., 2023). While our knowledge about the role of the gut virome (GV) in health and disease is still in its early stages (Iorio et al., 2022), evidence has emerged that this kingdom, too, is disrupted upon SARS-CoV-2 infection. Studies in adult patients have found the GV of affected patients to be dysbiotic in a way that was proportional to disease severity (Cao et al., 2021; Zuo et al., 2021b; Karim et al., 2023). However, the role of the GV in COVID-19 progression remains to be fully elucidated, and in children it still remains to be described at all.

Recently, our group found that, unlike adults, *F. prausnitzii* is enriched in the GM of pediatric COVID-19 patients, which may contribute to their protection against the more severe manifestations of the disease (Romani et al., 2022). Here, we provide a more in-depth analysis of GM ecology and functional profiling of a subset of these pediatric patients by shotgun metagenomics, to investigate the bacterial composition of their GM at the species level, as well as the first description of the pediatric GV in response to SARS-CoV-2 infection. Furthermore, we perform a functional analysis of our metagenomics data, in order to elucidate and the effect of both SARS-CoV-2 infection and preventative antibiotics use on antimicrobial resistance (AMR) and virulence gene prevalence.

## Materials and methods

### Patient selection

Children admitted to the Bambino Gesù Children’s Hospital with symptoms related to COVID-19 between March and September 2020 were enrolled in this study. SARS-CoV-2 infection was confirmed *via* both nasopharyngeal swabs and subsequent reverse transcriptase qualitative polymerase chain reaction (PCR). This study was approved by the local ethics committee (2083_OPBG_2020) and informed consent was obtained from each of the children’s parents or legal guardians. Routine clinical characteristics and stool samples were collected close to the time of their admission. Stool samples were then stocked at -80°C for later nucleic acid extraction and metagenomic sequencing.

### Nucleic acid extraction

DNA was extracted from stool samples with a QIAamp PowerFecal Pro DNA Kit (Qiagen) according to the manufacturer’s instructions. Briefly, approx 50mg of stool and 800 µl of CD1 Buffer was added to a tube containing glass beads and vortexed for 20 mins. Inhibitors were removed and DNA was bound with CD2 and CD3 Buffer, respectively, and the solution was then loaded onto the column. The column was washed with EA and C5 Solution, then the DNA was eluted and the concentration was determined with PicoGreen. The DNA was then stored at -20°C for later use.

### Shotgun metagenomic sequencing

Shotgun metagenomic sequencing libraries were prepared with Illumina’s Tagmentation Protocol with the Illumina DNA Prep Kit as per the manufacturer’s instructions. DNA fragments were then barcoded with Nextera™ DNA CD Indexes (Illumina). Library concentrations were determined with PicoGreen and samples were pooled for sequencing. Pooled libraries were sequenced on a NextSeq 500 sequencing platform (Illumina).

### Microbial profiling and functional analysis

Paired-end sequencing reads were imported into CLC Genomics Workbench (Qiagen) and submitted to quality controls, whereby the reads were trimmed (trimmed from 3’ end; quality limit 0.05; ambiguous nucleotides trimmed; maximum number of ambiguities 2) and low-quality reads were discarded. Bacterial and viral taxonomic assignment was determined with the Microbial Genomics Module (match score 1; mismatch cost 2) by interrogating the Unified Human Gastrointestinal Genome (UHGG) Database and the Clustered Reference Viral DataBase (RVDB), respectively. Reads from the host were filtered and removed by interrogating the *Homo sapiens* GRCh38 sequence. For functional analyses, sequencing reads were assembled into longer contigs with the *de novo* Metagenome Assembly tool in the Microbial Genomics Module (minimum contig length: 200bp) by iterating the assembler three times with increasing wordsize (*k* = 21, 41, 61). Antimicrobial resistance and virulence genes present in these contigs were identified with the Nucleotide DB tool (Zankari et al., 2017) (minimum identity 98%; minimum length 60%) by interrogating the Qiagen Microbial Insight Antimicrobial Resistance (QMI-AR) and Virulence Factor DataBase (VFDB), respectively. Statistical Analyses were performed within the CLC Genomics Workbench software using multi-factorial statistics based on a negative binomial Generalized Linear Model (GLM) and all results were controlled for age bracket.

## Results

### Study cohort

This study was performed on a subset of samples from patients with confirmed COVID-19 diagnosis who had visited the Bambino Gesù Children’s Hospital in Rome, Italy, and who had been enrolled for a previously published study (Romani et al., 2022). Patients under the age of 6 years were excluded due to the rapidly evolving and highly variable state of the GM during this developmental stage (Roswall et al., 2021). Patients were thus divided into two age brackets: pre-teen (7-12 years of age) and teenagers (13-18 years of age). Patients with comorbidities and known co-infections were also excluded from this dataset to limit confounding factors. Finally, 22 patients with COVID-19 were analyzed for this study, 7 of whom had been administered antibiotics before stool sample collection and 15 of whom had not, and results were compared with samples from 22 age- and sex-matched healthy children (**Table 1**).

**Table 1:** Patient demographics and clinical manifestations at admission.

Variables	COVID-19	Healthy Controls
Number of subjects	22	22
Male	11	10
Female	11	12
Median Age (IQR)	13 (9.25 – 14.75)	11 (10 – 13)
Symptoms (%)		
Fever	14 (63.64%)	
Cough	7 (31.82%)	
Shortness of Breath	4 (18.18%)	
Diarrhea	4 (18.18%)	
Abdominal Pain	1 (4.55%)	
Headache	2 (9.09%)	
Myalgia	4 (18.18%)	
Seizure	1 (4.55%)	
Pharyngitis	1 (4.55%)	
Rash	1 (4.55%)	
Chest Involvement (X-ray)	7 (31.82%)	
Ileum and colon thickening	1 (4.55%)	
Antibiotics (single dose)	4 (18.18%)	
Antibiotics (full course)	3 (13.64%)	

### SARS-CoV 2 infection causes significant shifts in gut bacterial taxa in pediatric patients

**Figure 1:**
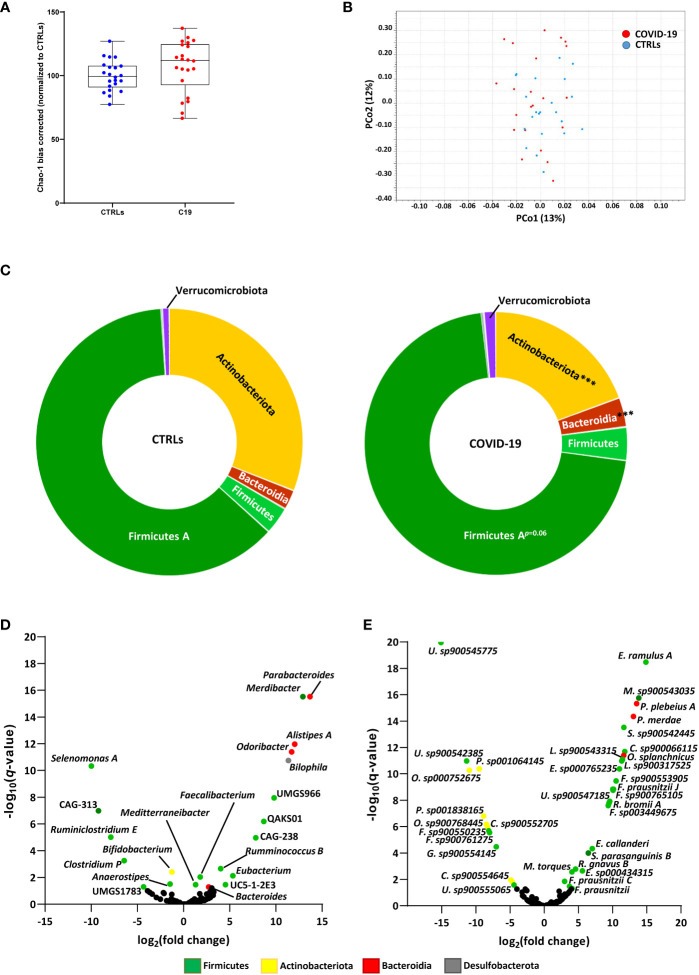
SARS-CoV-2 infection leads to altered bacterial composition in the gut. **(A, B)** Alpha diversity, as measured by the bias corrected Chao-1 analysis **(A)**, and beta-diversity by Bray-Curtis analysis **(B)** of COVID-19 pediatric patients (red) and healthy subjects (CTRLs, blue). Alpha diversity measurements expressed as percentages (normalized to the mean of the CTRLs). **(C)** Relative distributions of bacterial phyla in COVID-19 (right) and CTRLs (left). Statistical analysis: negative binomial Generalized Linear Model (GLM) corrected for age bracket FDR. ***q<0.001. **(D, E)** Volcano plot of differentially abundant bacterial genera **(D)** and species **(E)** in COVID-19 patients compared to CTRLs. Datapoints color coded by phylum. In black: genera and species which were not significantly differentially abundant.

First, we analyzed the bacterial composition of the GM of children infected with SARS-CoV 2, who were enrolled in the study before vaccines against SARS-CoV-2 were made widely available. There were no significant differences in alpha diversity, as measured by the Chao-1 (bias corrected) method (**Figure 1A**), or beta diversity, as measured by Bray-Curtis (**Figure 1B**), Jaccard and Euclidean methods (data not shown). However, we did find differences in the proportions of specific bacteria at the phylum level. SARS-CoV-2-infected patients had significantly reduced proportions of Actinobacteriota and increased proportions of Bacteroidia compared to healthy age-matched controls (CTRLs), with a consistent expansion of Firmicutes A which just failed to reach significance after FDR correction (**Figure 1C**). Within these phyla, 21 genera and 35 species were differentially abundant in patients with COVID-19 compared to CTRLs (**Figures 1D, E**).

Consistent with previous findings in adults, we found a significant decrease in *Bifidobacterium* and an over-abundance of *Parabacteroides, Odoribacter* and *Ruminococcus gnavus*, the latter having also been previously associated with inflammatory bowel diseases (IBD) (Gaibani et al., 2021; Yeoh et al., 2021; Wang et al., 2023). Contrasting with data from adults but consistent with previous findings in pediatric patients (Romani et al., 2022), we also found an enrichment of the *Faecalibacterium* genus and several *Faecalibacterium prausnitzii* subspecies in our COVID-19 patient cohort (**Figures 1D, E**). Furthermore, the *Eubacterium* genus, whose abundance in the GM has previously been found to be inversely correlated with two of the inflammatory cytokines responsible for COVID-19 severity (Yeoh et al., 2021), was also enriched in our pediatric patients compared with CTRLs (**Figure 1D**). Similarly, we found an abundance of the *Alistipes* genus, which has also been inversely correlated with SARS-CoV-2 viral load (Wang et al., 2023). Taken together, these results indicate that SARS-CoV-2 affects the GM composition of pediatric patients, by enriching both pro- and anti-inflammatory bacteria.

### Treatment with antibiotics has a partial confounding effect on the GM of COVID-19 patients

During the pandemic, there was a marked increase in antibiotics treatment, at times due to a fear of the development of dangerous co-infections, while at others due to an initial misdiagnosis of the illness at hand (Stoichitoiu et al., 2022). Consistently, in our patient cohort, 7 out of the 22 COVID-19 patients were given antibiotics before their samples were collected for metagenomic sequencing. In 4 of these cases, the patient had only received a single dose of antibiotics in the 24 hours before stool samples were collected for GM profiling, while in the other 3, the patients had completed a full course of antibiotics immediately before the beginning of the study (**Table 1**). In order to see what effect antibiotics may have had on the GM of our COVID-19 patients, we decided to investigate the trends in GM ecology in patients who had received a single dose of antibiotics (C19^s.dose^) and patients who had received a full course of antibiotics treatment (C19^full.t^) compared to COVID-19 patients who had not been treated with antibiotics and controls (C19 and CTRLs, respectively).

**Figure 2:**
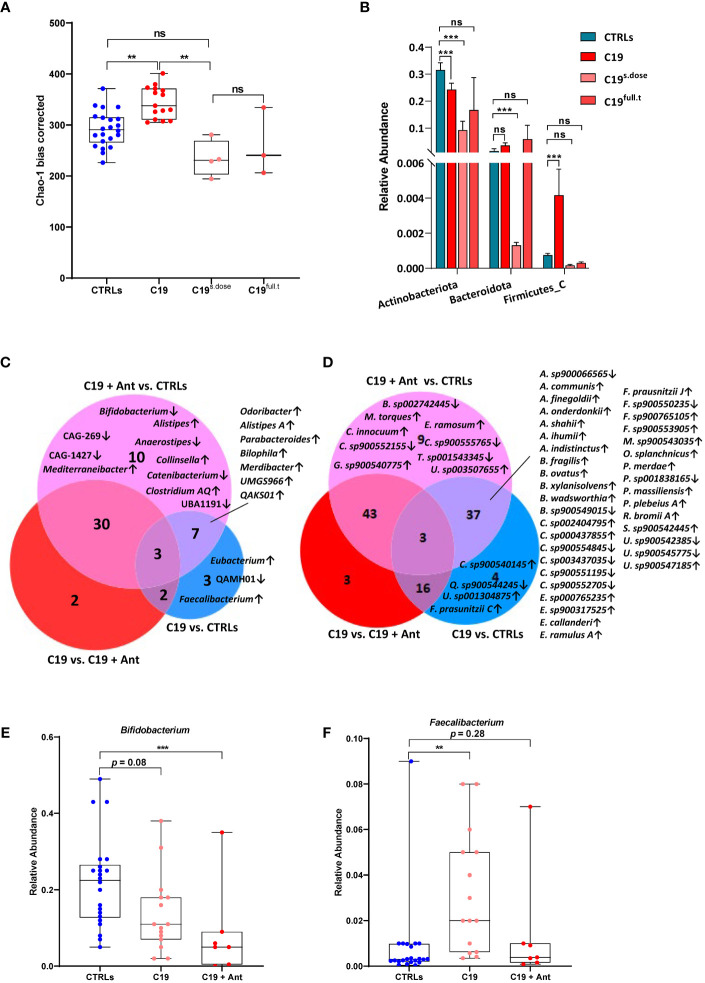
Effect of antibiotics use on gut dysbiosis in COVID-19 pediatric patients. **(A)** Alpha diversity, as measured by the bias corrected Chao-1 analysis of COVID-19 pediatric patients treated with a single dose of antibiotics within 24 hours of sample collection (C19^s.dose^), patients treated with a full of antibiotics before sample collection (C19^full.t^), untreated patients (C19) and healthy subjects (CTRLs). **(B)** Relative abundance of significantly differentially abundant bacterial phyla. **(C, D)** Venn Diagram of bacterial genera **(C)** and species **(D)** found to be significantly differentially abundant between CTRLs and COVID-19 patients (CTRLS *vs*. C19), CTRLs and COVID-19 patients treated with antibiotics (CTRLs *vs* C19 + Ant) and COVID-19 patients compared to COVID-19 patients treated with antibiotics (C19 *vs* C19 + Ant). Arrows indicate whether the genera/species are more (up) or less (down) abundant, compared to CTRLs. **(E, F)** Box plots of the relative abundance of *Bifidobacterium* **(E)** and *Faecalibacterium* **(F)** in CTRLs, untreated COVID-19 patients (C19) and COVID-19 patients treated with antibiotics (C19 + Ant). Statistical analysis for B-D: negative binomial Generalized Linear Model (GLM) corrected for age bracket and FDR. **p=0.01; ***p<0.001; ns, not significant.

We found that, while alpha diversity was not significantly affected when the COVID-19 patients were taken as a whole (**Figure 1A**), significant differences were observed when we included antibiotics use as a variable. In our cohort, COVID-19 patients who did not receive antibiotics actually had an increase in alpha-diversity compared to CTRLs, which was decreased in patients receiving antibiotics (**Figure 2A**). Interestingly, even a single dose of antibiotics taken within 24 hours of stool sample collection was enough to cause a significant shift in bacterial alpha diversity in these patients (**Figure 2A**). On the other hand, antibiotics use did not lead to a shift in the beta-diversity of the GM of these patients (data not shown). Given the fact that even a single dose of antibiotics had a similar effect on bacterial alpha diversity as did treatment with a full course of antibiotics, and given that the number of treated patients was very small, we next decided to pool all treated patients into a single group (C19 + Ant) to see how antibiotics treatment affected the abundance of specific bacteria within the GM of COVID-19 patients.

At the taxonomic level, antibiotics had contrasting effects on the relative abundances of bacterial phyla (**Figure 2B**). In the case of Actinobacteriota and Firmicutes A, antibiotics use exacerbated the effect of SARS-CoV-2 infection, by either further decreasing or further increasing their relative abundances (**Figure 2B**). In others, such as with Firmicutes C, antibiotics seemed to reverse the effect of SARS-CoV 2 infection, with relative abundances that more closely resembled those of CTRLs (**Figure 2B**). Therefore, we decided to investigate which bacterial genera and species were differentially abundant either dependently or independently of antibiotics use.

We compared which bacterial genera (**Figure 2C**) and species (**Figure 2D**) were significantly differentially abundant in C19 *versus* CTRLs, C19 + Ant *versus* CTRLs, and C19 *versus* C19 + Ant. No genera were found to be significantly differentially enriched in all three groups (**Figure 2C**). On the other hand, a decrease in the *Bifidobacterium* genus was only found to be significantly decreased in the C19 + Ant group compared to CTRLs (**Figure 2C**). Upon closer inspection, it seems that, while the C19 group does have a decrease in the *Bifidobacterium* genus, as previously reported, antibiotics further exacerbate this decrease (**Figure 2E**). Conversely, the *Faecalibacterium* genus was only significantly increased in the C19 group compared to CTRLs (**Figure 2C**). Once again, upon closer inspection, it seems that antibiotics use contrasts the enrichment of *Faecalibacterium* normally found in pediatric patients with COVID-19 (**Figure 2F**). Taken together, these results suggest that treating patients with antibiotics can lead to the reduction of potentially protective bacteria in the GM of COVID-19 patients.

Similarly, when we compare these groups at the species level, the subspecies *F. prausnitzii C* was exclusively enriched in the C19 group. However, *F. prausnitzii J* was enriched in both the C19 and the C19 + Ant groups compared to CTRLs. In fact, when analyzed at the species level, many reported bacterial signatures were significantly represented in both the C19 and C19 + Ant groups, indicating that the use of antibiotics did not alter many of the microbial signatures associated with COVID-19. Specifically, species correlated with low COVID-19 severity such as *Parabacteroides merdae, Bacteroides ovatus, Alistipes onderdonkii* and *Alistipes shahii*, were all significantly enriched in both C19 and C19 + Ant groups compared to CTRLs (**Figure 2D**). On the other hand, other species correlated with intestinal inflammation, such as *Alistipes finegoldii*, and *Bacteroides fragilis*, were also enriched in both C19 and C19 + Ant, compared to CTRLs (**Figure 2D**). Taken together, these results indicate that antibiotics use had only a partial effect on the GM of COVID-19 patients, as many of the reported bacterial signatures associated with SARS-CoV-2 infection remain present.

### The gut virome is also partially affected by antibiotics use in COVID-19 patients

Given the interconnected relationship between bacteria and the other micro-organisms that share their ecological niche, we next decided to investigate the virome of these patients, as they are the next most abundant and diverse microbial population of the GM.

**Figure 3:**
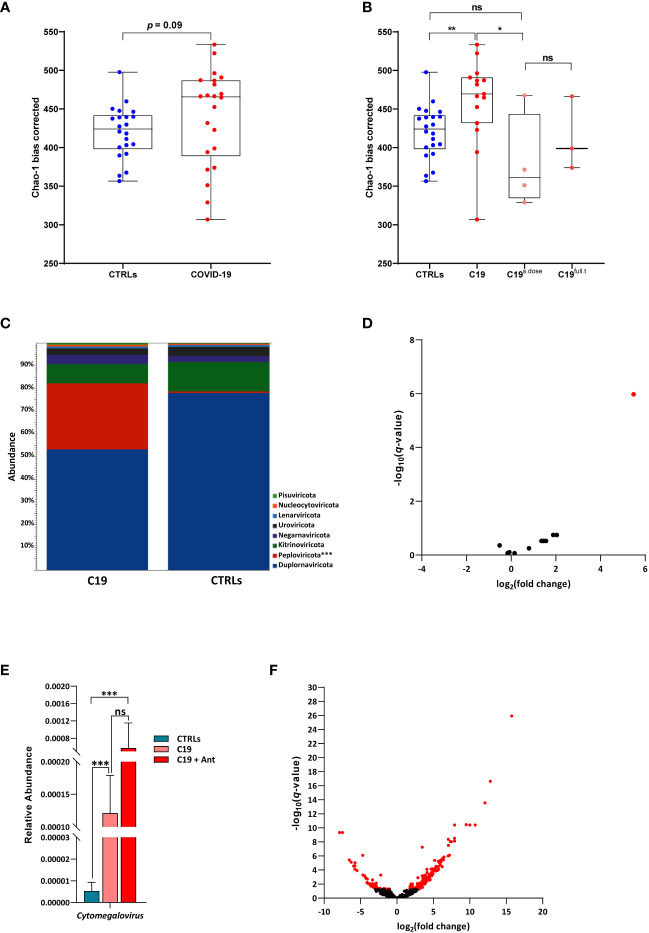
The pediatric gut virome in response to SARS-CoV-2 infection. **(A)** Alpha diversity, as measured by the bias corrected Chao-1 analysis of COVID-19 pediatric patients compared to CTRLs. **(B)** Alpha diversity, as measured by the bias corrected Chao-1 analysis of COVID-19 pediatric patients treated with a single dose of antibiotics within 24 hours of sample collection (C19^s.dose^), patients treated with a full of antibiotics before sample collection (C19^full.t^), untreated patients (C19) and healthy subjects (CTRLs). **(C)** Relative abundances of viral phyla in COVID-19 patients (left) compared to CTRLs (right). **(D)** Volcano plot of classified viral genera in COVID-19 patients compared to CTRLs. In red: significantly differentially abundant genera. **(E)** Relative abundance (normalized to mean CTLR value) of *Cytomegalovirus* in CTRLs, untreated COVID-19 patients (C19) and COVID-19 patients treated with antibiotics (C19 + Ant). **(F)** Volcano plot of all viruses and VLPs in COVID-19 patients compared to CTRLs. In red: significantly differentially abundant viruses/VLPs. *p=0.05; **p=0.01; **p=0.001; ns=not significant.

As with the bacterial diversity of our COVID-19 patients, we found no significant difference in viral alpha-diversity between patients and controls when our COVID-19 cohort was taken as a whole (**Figure 3A**). However, interestingly, we also observed a similar pattern as we did in our bacterial community after taking antibiotics use into account, despite the fact that antibiotics do not have a direct effect on viruses. Though the effect was less pronounced, we found that SARS-CoV-2 infection led to a significant increase in viral alpha diversity when compared to CTRLs, which was reversed in COVID-19 patients treated with antibiotics (**Figure 3B**), indicating that the viral community is highly responsive to the bacterial one within the intestine.

When we focused our analysis only on classified viruses identified in our patients, we found that there were no significant differences between the C19 and C19 + Ant groups at the phylum level, with only a marked increase in the Peploviricota phylum found in COVID-19 patients compared to CTRLs (**Figure 3C**). At the genus level, we found an enrichment of the opportunistic pathogenic *Cytomegalovirus* genus in COVID-19 patients compared to CTRLs (**Figure 3D**). Furthermore, antibiotics use led to an even larger increase in *Cytomegalovirus* abundance (**Figure 3E**), indicating that antibiotics use can potentially intensify the presence of pathogenic viruses in the gut.

We next decided to expand our analysis to include all viruses and VLPs in our dataset, regardless of whether not we have taxonomic information about them. When all unclassified viruses and VLPs were taken into account, we observed that only 49 VLPs were significantly decreased, while 157 were significantly enriched in COVID-19 patients compared to CTRLs (**Figure 3F**). This marked increase in VLPs in COVID-19 patients was not matched by a consistent increase in species richness of bacteria across the entire COVID-19 patient group (**Figure 1A**), indicating that this increase is a signature of SARS-CoV-2 infection rather than an indirect effect derived from an increase in microbial species richness.

**Figure 4:**
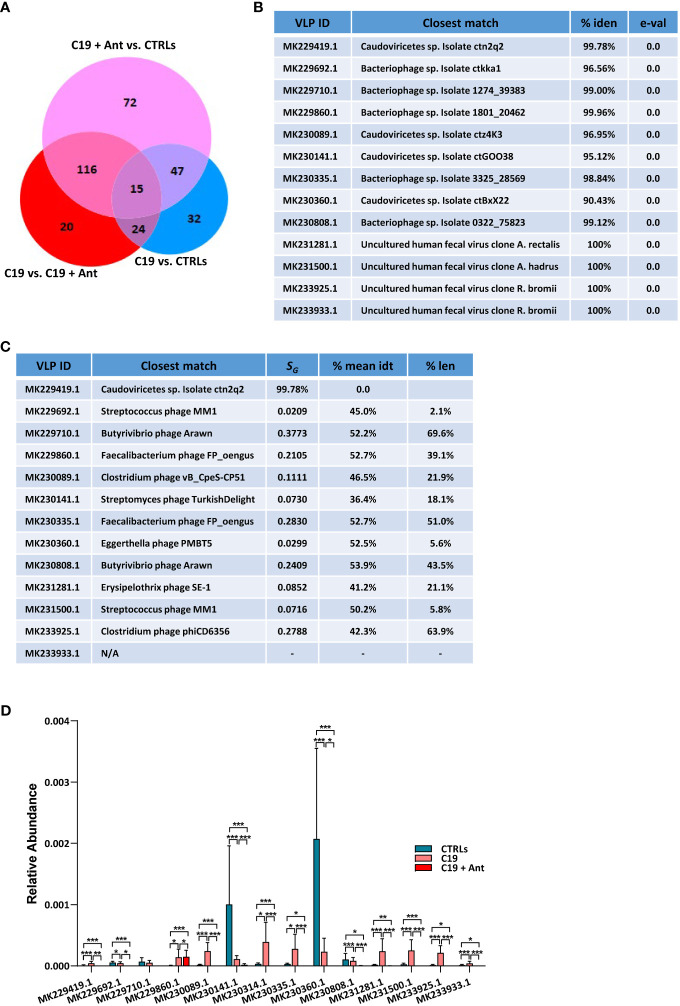
Antibiotics use does not have a universal effect on the relative abundance of VLPs in the GM of pediatric COVID-19 patients. **(A)** Venn Diagram of viruses and VLPs found to be significantly differentially abundant between CTRLs and COVID-19 patients (CTRLS *vs*. C19), CTRLs and COVID-19 patients treated with antibiotics (CTRLs *vs* C19 + Ant) and COVID-19 patients compared to COVID-19 patients treated with antibiotics (C19 *vs* C19 + Ant). **(B)** Table reporting the results of searching those 14 unclassified VLPs with megaBLAST against reference viral databases in NCBI, including the name of the virus with the closest sequence identity, the percentage of sequence identity between the two, and the reported e-value. **(C)** Table of the closest viral match by phylogenetic analysis using VipTree (v4.0). *S_G_*: genomic similarity score. **(D)** Relative abundances of the 14 unclassified VLPs in CTRLs, untreated COVID-19 patients (C19) and COVID-19 patients treated with antibiotics (C19 + Ant). Statistical analysis for **A, C**: negative binomial Generalized Linear Model (GLM) corrected for age bracket and FDR. *p<0.05; **p<0.01; ***p<0.001; ns, not significant.

When taking antibiotics into account, we found that 15 VLPs were significantly differentially enriched in C19 *versus* CTRLs, C19 + Ant *versus* CTRLs, and C19 *versus* C19 + Ant (**Figure 4A**), one of which was *Cytomegalovirus* (CMV), while the other 14 were unclassified VLPs (**Figures 4B, C**). In order to obtain more information about these unclassified VLPs, we next performed a megaBLAST search with each of these sequences, to see with which viruses they shared the most sequence identity. We found that all of the VLPs that were significantly differentially abundant in our three groups were bacteriophages (**Figures 4B, C**).

Next, we decided to see whether antibiotics use had a consistent pattern on the relative abundance of these 14 VLPs, presumably bacteriophages. We found that antibiotics use did not lead to an overall further increase in the abundance of the presumably commensal VLPs as it did with the opportunistic pathogen CMV. In 8 out of the 9 C19 cases where SARS-CoV-2 infection led to an increase in the abundance of a VLP, C19 + Ant actually had a reduction in those same VLPs, leading to a relative abundance that more closely resembled that of CTLRs (**Figure 4D**). In those 4 cases where COVID-19 led to a reduction in relative abundance of VLPs, on the other hand, patients who had taken antibiotics showed a further reduction in the relative abundance of those same VLPs (**Figure 4D**). Only in one of those cases, namely MK229860.1, did antibiotics lead to a further increased abundance compared to untreated COVID-19 patients, and even in that case the difference was slight (**Figure 4C**). Therefore, as a whole, antibiotics use led to a reduction in the relative abundance of commensal viruses in the pediatric GM.

### SARS-CoV-2 infection leads to the altered abundance of antimicrobial resistance genes

The human gut can act as a reservoir of antimicrobial resistance (AMR) genes which, when perturbed, can lead to the rise of AMR-carrying pathobionts (Anthony et al., 2021). We therefore decided to investigate whether or not COVID-19 infection could lead to the differential enrichment of AMR genes in the GM of pediatric patients. To this end, we interrogated the curated QMI-AR database, which combines AMR peptide markers from the Comprehensive Antibiotic Resistance Database (CARD), Antibiotic Resistence Gene-ANNOTation (ARG-ANNOT), the NCBI Bacterial Antimicrobial Resistance Gene Database and ResFinder.

**Figure 5:**
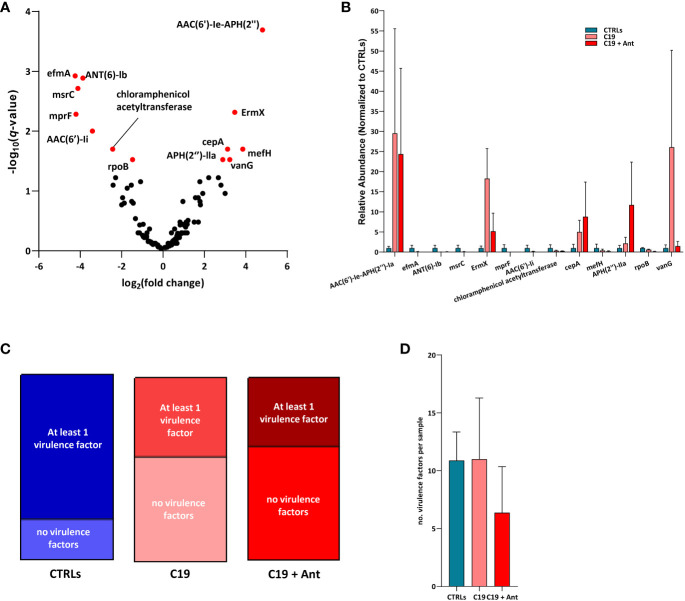
SARS-CoV-2 infection leads to shifts in the abundance of genes encoding for antimicrobial resistance, but not for virulence factors. **(A)** Volcano plot of AMR gene abundance in COVID-19 patients compared to CTRLs. In red: significantly differentially abundant genes. Statistical analysis: negative binomial Generalized Linear Model (GLM) corrected for age bracket and FDR **(B)** Bar chart of the relative abundances of the 13 differentially abundant genes in CTRLs (blue), untreated COVID-19 patients (C19, pink) and COVID-19 patients treated with antibiotics (C19 + Ant, red). **(C)** Proportions of CTRL (left, blue), untreated COVID-19 (middle, pink) and antibiotics treated COVID-19 (right, red) patients in which virulence factors were detected. **(D)** Average number of virulence factors found in each CTRL (blue), untreated COVID-19 (C19, pink) and antibiotics treated (C19 + Ant, red) patient.

We found that, in our dataset, 6 AMR genes were significantly enriched and 7 were significantly reduced in COVID-19 patients compared with CTRLs, indicating that SARS-CoV-2 infection can indeed lead to shifts in AMR in pediatric patients (**Figure 5A**). Four of the genes found to be differentially abundant in our dataset are genes that confer resistance to aminoglycoside antibiotics. Two of these, the aminoglycoside phosphotransferase *APH(2’’)-IIa*, and the aminoglycoside acetyltransferase *AAC(6’)-Ie-APH(2’’)*, were more abundant in COVID-19 patients compared to CTRLs (**Figure 5A**). Meanwhile, the aminoglycoside nucleotidyltransferase *ANT (6)-Ib*, and the aminoglycoside acetyltransferase *AAC(6’)-Ii* were significantly reduced in COVID-19 patients. Similarly, the rRNA methyltransferase *ErmX*, which confers resistance to streptogramin antibiotics, was significantly enriched in COVID-19 patients, while the *Enterococcus faecium*-derived *msrC*, which also confers resistance to streptogramin antibiotics, was significantly reduced (**Figure 5A**).

On the other hand, the *Bacteroides fragilis*-derived beta-lactamase *cepA*, which hydrolyzes cephalosporin (Rogers et al., 1994), and the vancomycin-resistance gene *vanG*, were both exclusively enriched in COVID-19 patients. Furthermore, the *Bifidobacterium*-derived beta-subunit of RNA polymerase *rpoB*, which confers resistance to rifamycins, chloramphenicol acetyltransferase, and the negatively charged phosphatidylglycerol *mprF*, which confers resistance to defensins, were both significantly reduced in COVID-19 patients (**Figure 5A**). Taken together, these results suggest that the gut dysbiosis induced by SARS-CoV-2 infection results in a consequent shift in the relative abundance of AMR genes.

### Treatment with antibiotics did not lead to an overall increase in AMR gene abundance

One of the greatest risks associated with the overuse of antibiotics is the consequent increase in prevalence of AMR genes. Given the effects of antibiotics use on the GM of COVID-19 patients described above, we decided to investigate whether this treatment also led to an increase in AMR in these patients. Interestingly, the addition of antibiotics treatment did not have a universal pattern of influence on the relative abundances of these AMR genes. In fact, of the 13 differentially abundant AMR genes in our dataset, only 2 were even more abundant in COVID-19 patients treated with antibiotics than they were in untreated children infected with SARS-CoV-2, and only one of which was significantly increased (**Figure 5B**). Antibiotics use led to a significant increase in the relative abundance of the aminoglycoside phosphotransferase *APH(2’’)-IIa*, and very slightly increased the relative abundance of the *Bacteroides fragilis*-derived beta-lactamase *cepA* (**Figure 5B**). Conversely, 10 out of the 11 remaining AMR genes found were substantially reduced in the C19 + Ant cohort, while the relative abundance of the last, the aminoglycoside acetyltransferase *AAC(6’)-Ie-APH(2’’)*, was largely unchanged in the group treated with antibiotics (**Figure 5B**). In all 7 of the AMR genes that were significantly reduced in COVID-19 patients, antibiotics treatment led to either no significant change or to a further reduction of the relative abundance of the gene (**Figure 5B**). Finally, 2 AMR genes which were significantly enriched in the C19 group, namely *ErmX* and *vanG*, antibiotics use reduced their relative abundance to more closely resemble CTRL levels (**Figure 5B**). These data suggest that, shortly following treatment with antibiotics, AMR genes do not, overall, rapidly become more enriched in the GM.

### SARS-CoV-2 infection does not increase virulence factors in the GM of affected pediatric patients

Apart from AMR, bacteria can also acquire genes which make them more virulent, which can also be an effective method to increase infectivity. Therefore, we decided to see whether or not SARS-CoV-2 infection, with or without antibiotics use, may have had an effect on the abundance of virulence factors in the GM. For this analysis, we assembled our sequencing reads into a contigs list for each patient sample, and used them to interrogate the Virulence Factor DataBase (VFDB), which contains both confirmed and hypothetical bacterial virulence factors.

SARS-CoV-2 infection did not increase the number of virulence factors in the GM of patients, but rather decreased the prevalence of these genes. While approximately 76% of CTRLs had at least one virulence factor in their GM contig list, under 40% of COVID-19 patients had any virulence factors in theirs, whether they had taken antibiotics or not (**Figure 5C**). Furthermore, there was no significant difference between the average number of virulence factors found between the three groups (**Figure 5D**). These results rather suggest that, if SARS-CoV-2 infection has any effect at all, it is to reduce the presence of virulence genes in the GM.

## Discussion

In this study, we performed an in-depth GM analysis of a subset of pediatric COVID-19 patients by shotgun metagenomics. Consistent with previous findings in adult cohorts (Gu et al., 2020; Zuo et al., 2020; Yeoh et al., 2021; Mizutani et al., 2022), our pediatric patient group had some pro-inflammatory GM signatures. For example, one of the most common COVID-related GM signatures is a decreased abundance of members of the *Bifidobacterium* genus, which we also observed in our patient group. Another very common signature in both intestinal inflammation in general and COVID-19 in particular is the increase of *Bacteroides fragilis*, which was also consistent in our dataset. Furthermore, we found *A. finegoldii* to be enriched in this dataset, which has been positively correlated with inflammatory biomarkers and was found to steadily increased in abundance as COVID-19 became more severe (Schult et al., 2022). However, unlike in adult patients (Tang et al., 2020; Zuo et al., 2020; Reinold et al., 2021; Yeoh et al., 2021) but consistent with our previous study in children (Romani et al., 2022), we found that *F. prausnitzii* was enriched in our pediatric COVID-19 patients. Given that *F. prausnitzii* abundance has been found to be negatively correlated with both SARS-CoV-2-induced proinflammatory cytokines (Mizutani et al., 2022) and with COVID-19 severity (Tang et al., 2020; Khan et al., 2021; Reinold et al., 2021), we hypothesize that its abundance in the pediatric GM could contribute to their protection against the more severe manifestations of the disease.

However, in this study, we find that *F. prausnitzii* is not the only protective bacterial species that we found to be enriched in our pediatric COVID-19 patients. For example, the genera *Bilophila* and *Parabacteroides*, which were both found to be negatively correlated with COVID-19 severity adults (Tao et al., 2020), were found to be enriched in our pediatric COVID-19 patients. Similarly, our patients were enriched for short chain fatty acid-producing members of the *Alistipes* genus, such as *A. onderdonkii* which, along with *P. merdae*, were found to be protective against intestinal inflammation and correlated with low SARS-CoV-2 infectivity (Zuo et al., 2021a). Furthermore *B. ovatus*, which has been shown to reduce colonic *ACE2* expression and showed negative correlation with faecal SARS-CoV-2 load in COVID-19 patients (Zuo et al., 2020), was also enriched in our pediatric COVID-19 patients. Taken together, these data suggest that the GM of children may possess a balance of multiple bacterial species that work in concert to reduce SARS-CoV-2 infectivity and severity.

Another way that our data differed substantially from results found in adults was in terms of species richness. Most studies conducted in adult patients found a significant reduction in alpha-diversity in the GM of COVID-19 patients (Gu et al., 2020; Gaibani et al., 2021; Wang et al., 2023), while others found no significant difference between COVID-19 patients and CTRLs (Mizutani et al., 2022). In pediatric patients, alpha diversity has been reported to be decreased in children infected with SARS-CoV-2 (Romani et al., 2022), or not significantly affected, though with a noticeable trend towards an increase in species richness (Suskun et al., 2022). These conflicting results may be due to a number of different factors, such as the method of metagenomics profiling, the selection criteria for healthy CTRLs, or whether or not antibiotics use is taken into consideration during the analysis.

In fact, in our patient cohort, we found no significant difference in alpha-diversity (**Figure 1A**) until we controlled for antibiotics use (**Figure 2A**). When we split our COVID-19 group into those who had been administered antibiotics and those who had not, we found that, in our cohort, alpha diversity was actually increased in our untreated COVID-19 patients, while it was significantly reduced in those who had received antibiotics. Interestingly, one study found that alpha diversity too was negatively correlated with COVID-19 severity and poor prognosis (Wang et al., 2023), suggesting another possible reason for the conflicting results reported in the literature. Therefore, it is possible that SARS-CoV-2 infection can have different effects on the alpha-diversity of the GM depending on how long the virus has infected the patient, and on how severe the infection is.

Initial reports indicated that severe SARS-CoV-2 infection could leave patients predisposed to secondary co-infections from opportunistic pathogens, particularly to bacterial pneumonia, which may have accounted for a portion of COVID-19-related deaths (Zuo et al., 2020; Fazel et al., 2023). Consequently, there was a marked increase in antibiotics use in 2020 compared to 2019, both in Italy and across the world (Hussein et al., 2022; Stoichitoiu et al., 2022; Perrella et al., 2023). In a hospital setting, especially at the beginning of the pandemic, many physicians prescribed antibiotics to their COVID-19 patients, sometimes as a result of an initial misdiagnosis of a bacterial respiratory infection, other times as a precautionary measure against hospital-acquired co-infections, especially in those with comorbidities or who were otherwise deemed to be high risk patients (Stoichitoiu et al., 2022). Indeed, those patients in our cohort who received antibiotics upon admission did so because, in the early days of the pandemic, preventative antibiotics use was indicated for patients whose COVID-19 infection was severe enough to produce symptoms. However, overuse of antibiotics can also lead to the depletion of protective bacteria in the gut, leading many to question whether the preventative use of antibiotics may have been counter-productive during COVID-19 treatment (Granata et al., 2022), which was why this practice was also abandoned in our hospital approximately six months after it was instated.

We found that antibiotics use did indeed have a substantial effect on the GM of COVID-19 patients, even after a single dose was administered within 24 hours of sample collection. We found that antibiotics not only reduced the alpha-diversity of the GM, but also led to the differential abundance of many bacterial genera and species, including protective ones. However, we also found that many of the pediatric GM signatures of COVID-19, including the protective ones such as *F. prausnitzii, B. ovatus* and *A. onderdonkii*, were consistent regardless of antibiotics use. Therefore, while antibiotics did negatively affect the GM to some extent, it was not enough to have an overall damaging effect on the abundance of the protective species present on the GM, at least in the short term. Further studies in pediatric patients are needed to see whether completing one or multiple rounds of antibiotics could further deplete these protective species, and, consequently, whether this may also predispose children to more severe manifestations of COVID-19.

Though the bacterial population of the human intestine is the most studied by far, the GM is actually a dynamic interconnected community of bacteria, viruses, archaea and eukaryotes, which each have their role to play in human health and disease. To this end, we decided to investigate the composition of the GV in these subjects, to see whether or not this community was also affected by SARS-CoV-2 infection. The only classified virus that we found to be differentially abundant in COVID-19 patients compared to CTRLs is CMV, an opportunistic pathogen which is commonly found persisting in a latent stage in the GM. However, intestinal inflammation, such as in IBD, can cause favorable conditions for CMV to replicate and exacerbate that inflammation (Jentzer et al., 2020). While the role of CMV in IBD is still a matter of debate, high fecal CMV load is nonetheless associated with intestinal inflammation, and can predispose a patient to CMV-colitis (Jentzer et al., 2020). Here, we found that SARS-CoV-2 increases CMV viral load in the GM of COVID-19 patients, which is yet another pro-inflammatory signature in the GM of these patients.

It is important to note that there is far less known about the GV is than there is of the bacteriome and, at the same time, viruses are far trickier to culture and characterize than their prokaryotic counterparts. Because of this, the majority of viruses and VLPs present in these available databases, such as the RVDB used in this study, remain uncharacterized and unclassified. Therefore, when we expanded our analysis to include all VLPs, we found hundreds of differentially abundant VLPs in COVID-19 patients compared with CTRLs. In particular, 157 VLPs were significantly increased, while 49 VLPs were significantly depleted in COVID-19 patients compared to controls, indicating that, on average, SARS-CoV-2 infection leads to an overall enrichment of viruses in the GM of affected patients.

Furthermore, while, by definition, antibiotics only target bacteria, we nevertheless wanted to see if antibiotics use would have an indirect effect on GV composition through its effect on intestinal bacteria. Interestingly, antibiotics also had an overall effect on the commensal virome of these patients. Similar to what we observed for the bacterial portion of the GM, antibiotics also led to a reduction of viral alpha-diversity in these patients. However, perhaps this is not as surprising as it may seem initially, given than many of the commensal viruses found in the GM are bacteriophages. Furthermore, these results are consistent with previous reports stating a correlation between the alpha diversity of the human gut bacteriome and virome (Pargin et al., 2023). Therefore, it very likely that this overall reduction of viral alpha-diversity is due to the reduction of the bacteriophages’ bacterial hosts.

When it came to CMV infection, we found that antibiotics use actually increased CMV viral load in COVID-19 patients. These results indicate that, when it comes to opportunistic viral pathogens, antibiotics may predispose patients to increased intestinal inflammation and/or viral co-infection, perhaps by decreasing bacterial diversity in the GM and thus causing more favorable conditions for pathogenic replication and colonization.

Given the effect of antibiotics on viral diversity in the gut, we next decided to see which VLPs were differentially abundant in our COVID-19 group split by antibiotics use. We found 15 VLPs that were significantly altered in all three groups. Interestingly, while antibiotic use was associated with an increase in CMV viral load, antibiotics actually led to a decrease in all but one of the VLPs that were differentially regulated in untreated COVID-19 patients compared to CTRLs. While this was initially surprising, further phylogenetic analysis of the affected VLPs revealed that they are most likely all bacteriophages, many of which are at least very closely related to the Caudoviricetes class. Caudoviricetes phages are among the most abundant in the GM, and their altered abundance has been associated with gut dysbiosis, birth mode, and disease. For example, increased Caudoviricetes abundance and decreased diversity has been associated with Crohn’s Disease, indicating that this phage class may have a role in intestinal inflammation (Pargin et al., 2023). While not enough is known about the role of these phages to conclude whether the signature we found in our patient cohort may be protective or damaging, we can conclude that SARS-CoV-2 infection is associated with a dysbiotic gut virome, particularly phages. Furthermore, we can conclude that antibiotics can also alter the balance of these phages independently of the effect of SARS-CoV-2 infection, likely by affecting their bacterial hosts.

AMR is one of the most pressing rising global health threats of our time. While the overprescription of antibiotics is undoubtedly the primary cause for this problem, it is undeniable that antibiotics cannot be fully dispensed with, leading to conflicting opinions as to when their prescription, especially for preventative purposes, may do more harm than good. Furthermore, other factors can also affect the prevalence of these genes, such as probiotics use and horizontal gene transfer. Therefore, we next decided to see whether or not the gut dysbiosis connected to SARS-CoV-2 infection also led to a consequent differential abundance of AMR genes. Surprisingly, there was no overall pattern of AMR increase in COVID-19 patients, whether treated with antibiotics or not. Instead, among the 13 differentially abundant AMR genes found in our dataset, 6 were found to be enriched while 7 were depleted in our COVID-19 patients, indicating a fairly even split between the two. Some of these genes, such as *rpoB* and *cepA*, followed the same abundance patterns as the bacterial species which produce them, namely *B. adolescentis* and *B. fragilis*, respectively. Other AMR genes found to be enriched in our dataset, on the other hand, have been attributed to multiple bacterial genera and species. For example, *vanG*, which confers protection against vancomycin, has been described in multiple different species, including known pathogens such as *Shigella dysenteriae*, *Salmonella enterica* and *Klebsiella pneumoniae*. However, *vanG* has also been described in the famously anti-inflammatory *Faecalibacterium prausnitzii*, several subspecies of which were also found to be significantly enriched in our COVID-19 patients (**Figure 2D**). However, *vanG* abundance in C19 + Ant patients was very similar to that found in CTRLs, while *F. prausnitzii* was consistently overrepresented across our COVID-19 patient cohort, regardless of antibiotics use (**Figure 2D**). These results are not consistent with the substantial decrease in *vanG* abundance observed in our C19 + Ant patients (**Figure 4B**). In light of these data, therefore, is it not possible to determine which bacteria are responsible for the observed abundance in *vanG* in our dataset, and may likely be caused by more than one species. What we can say is that antibiotics did not cause an overall increase in AMR genes in our patients, leading us to conclude that preventative AMR use in our pediatric COVID-19 patients did not result in an immediate detrimental response from the GM. Further studies are needed to confirm how many rounds of antibiotics would be necessary to lead to an appreciable increase in AMR in the GMs of pediatric COVID-19 patients, and whether this increase is an appropriate fear for preventative use of antibiotics in the treatment of COVID-19.

Opportunistic pathogens can become increasingly infective and problematic in two ways, either by evading treatment by developing AMR, or by increasing their virulence, thus increasing their infective potential. Therefore, we decided to also analyze our dataset for virulence factors, to see whether these were also altered in response to COVID-19 infection. However, to our surprise, we found that, if anything, SARS-CoV-2 infection led to a reduction in the prevalence of virulence factors in the GM. While we found at least one virulence factor in the GMs of 76% of CTRLs, we found at least one virulence factor in less than 40% of our COVID-19 patients, regardless of whether or not they had been treated with antibiotics. Furthermore, there was no significant difference in the number of virulence factor found in each patient. Considering the fact that the C19 group had the highest alpha-diversity of the three (**Figure 2A**), one might expect a slight increase in the number of any category of bacterial genes in to be found in this group, by virtue of probability. Instead, substantially fewer patients in both the C19 and C19 + Ant groups possessed any virulence factors in their GMs with respect to CTRLs, indicating that these results are completely uncoupled from species richness within the GM. These results suggest that COVID-19 may actually reduce the virulence of the GM, though such a hypothesis would need to be confirmed in a larger cohort of patients.

Our results further confirm that, in pediatric COVID-19 patients, there is a decidedly unique GM signature, prevalent in pro-inflammatory bacteria, opportunistic pathogens, but also protective, immunomodulatory bacteria that have been associated with low SARS-CoV-2 infectivity, mild symptoms, and good prognosis. Furthermore, in the short term, antibiotics seem to have a moderate effect on the GM, but do not immediately deplete these protective bacteria, though their use needs to be further validated with respect to duration of use and long-term effects. Our findings further underscore the value of the pediatric GM as an extremely valuable source of information for ways to combat severe COVID-19.

## Data availability statement

The datasets presented in this study can be found in online repositories. The names of the repository/repositories and accession number(s) can be found below: https://www.ncbi.nlm.nih.gov/, PRJNA1036590.

## Ethics statement

The studies involving humans were approved by Ethical Committee of the Bambino Gesù Children’s Hospital, IRCCS. The studies were conducted in accordance with the local legislation and institutional requirements. Written informed consent for participation in this study was provided by the participants’ legal guardians/next of kin.

## Author contributions

AP: Conceptualization, Formal analysis, Investigation, Methodology, Validation, Visualization, Writing – original draft. SP: Resources, Validation, Writing – review & editing. FD: Conceptualization, Resources, Writing – review & editing. LR: Resources, Writing – review & editing. AC: Resources, Writing – review & editing. PP: Funding acquisition, Resources, Supervision, Writing – review & editing. LP: Conceptualization, Funding acquisition, Resources, Supervision, Writing – original draft.

## Group member of CACTUS study team

Stefania Bernardi, MD, Francesca Calo` Carducci, MD, Caterina Cancrini, MD, PhD, Sara Chiurchiù, MD, Marta Ciofi degli Atti, MD, Nicola Cotugno, MD, Laura Cursi, MD, Renato Cutrera, MD, Carmen D’Amore, MD, Patrizia D’Argenio, MD, Maria A. De Ioris, MD, Maia De Luca, MD, Carlo Federico Perno, Prof., Andrea Finocchi, MD, PhD, Laura Lancella, MD, Giulia Linardos, MD, Emma Concetta Manno MD, Elena Morrocchi, PhD, Paola Pansa, MD, Libera Sessa, PhD, Alberto Villani, Prof., Paola Zangari, MD.
